# Treatment of coronary bifurcation lesions: stent-covering of the side branch with and without PCI of the side branch: a retrospective analysis of all consecutive patients

**DOI:** 10.1186/1471-2261-13-27

**Published:** 2013-04-04

**Authors:** Hubertus von Korn, Victor Stefan, Reyn van Ewijk, Kamalesh Chakraborty, Burkhard Sanwald, R Andel, Jan Hemker, Ulrich Hink, Marc Ohlow, Bernward Lauer, Thomas Muenzel

**Affiliations:** 1 Hetzelstift, Department of Cardiology, Stiftstr. 10, 67434 Neustadt, Germany; 2 IMBEI, University Medical Center Mainz, Obere Zahlbacher Str. 69, 55131 Mainz, Germany; 3 Department of Cardiology, University Hospital Mainz, Langenbeckstr. 1, 55131 Mainz, Germany; 4 Zentralklinik Bad Berka, Department of Cardiology, Robert-Koch-Allee 9, 99437 Bad Berka, Germany

**Keywords:** Coronary bifurcation lesions, PCI side branch, Simple vs complex strategy

## Abstract

**Background:**

Treatment of coronary bifurcation lesions is a complex problem.

**Methods:**

This retrospective single-center study included all consecutive patients with PCI of coronary bifurcations with stent covering of the side branch (SB) between January 2008 - August 2011.

Two methods were compared: group A represented patients without treatment of SB, group B were patients with treatment of SB.

**Results:**

Our study group (n = 98) was group A (n = 64, 65.3%) and group B (n = 34, 34.7%). Mean follow-up was 14.1 (group A) vs 12.3 (group B, p = ns) months.

Mean age (years) was 70.3 (group A) vs. 67.0 (group B, p = ns), NSTEMI/STEMI was present in 54.7% (group A) vs. 41,2% (group B, p = ns).

Duration of x-raying (min, group A vs group B) and the amount of contrast medium (ml) were significantly lower in group A: 18.1 min vs 20.1 min and 225.8 ml vs 307.4 ml (p < 0.05).

Final TIMI flow III inside the MB was reached in 98.4% (group A) vs. 97.1% (group B, p = ns), inside the SB in 84.4% vs. 94.1% (p = ns).

Target lesion revascularization and target vessel revascularization was seen in 15.9% (group A) vs 32.4% (group B, p = 0.07), cardiac death in 7.9% (group A) vs 14.7% (group B, p = 0.3).

All MACE revealed were: 23.8% (group A) vs. 47.1% (group B, p = 0.02).

**Conclusion:**

In patients with coronary bifurcations a simpler strategy has a significantly lower MACE.

**Trial registration:**

ClinicalTrials.gov Identifier: NCT01538186

## Background

The treatment of coronary bifurcation lesions is still a point of fervent discussion within the cardiologic community. Various treatment strategies are used where simple modalities are differentiated from more complex strategies.

The more complex treatment modalities include strategies where two stent are used [1-3], whereas a regular one-stent techniques is usually referred to as simple strategy.

Recent studies [4-7] have shown that a simpler strategy with stenting of only the main branch offers a better outcome than using two stents.

For this reason the provisional side branch stenting strategy has emerged as the preferred bifurcation treatment strategy.

We evaluated clinical outcome in a non-randomized registry of patients with coronary bifurcation lesions where the side branch had been covered with a stent. We then assigned two groups of patients as having undergone a PCI of the side branch or not before the clinical follow-up data was assessed.

## Methods

This retrospective observational study collected informations from all patients who underwent percutaneous treatment of a de novo coronary bifurcation lesion in a single German center between January 2008 and August 2011. We included all consecutively presenting patients with a bifurcation lesion where the side branch had been covered with a stent placed inside the main branch.

The bifurcation lesions were defined according to the Medina classification [8].

The study population included male or female patients older than 18 years of age with a diagnosis of stable angina or silent ischemia. Patients with an acute coronary syndrome (unstable angina, NSTEMI, STEMI, cardiogenic shock) were not excluded. Patients with a primary occlusion of the target vessel and a significant bifurcation lesion visible after thrombectomy were also kept in the patient collective.

The following patients were excluded for methodological reasons: patients with an in-stent-restenosis, patients with a therapy using a drug-coated balloon during the procedure (inside the main branch and/or the side branch) and patients where the side branch had not been covered by the stent inside the main branch.

Two different treatment strategies were compared: group A represented patients with a simple strategy without any treatment of the side branch (balloon angioplasty or stenting). Group B consisted of patients where the operator treated the side branch (balloon angioplasty and/or stenting) after or before stenting of the main branch following the concept of “provisional stenting”.

The demographic data, patient history, coronary risk factors, lesion location, morphology and procedural strategy were all documented.

For all patients we used a systematic approach for treating patients with coronary bifurcation lesions. This standard was established before the initiation of this registry. This standard is described in the chapter “angiographic procedure”.

The study complied with the Declaration of Helsinki regarding research on humans. All patients provided their written informed consent. An approval of an ethics committee was not intended due to the retrospective nature of our study.

### Angiographic procedure

Patients with an acute coronary syndrome were treated with aspirin 500 mg intravenously and 5000 IE heparin before admission to our hospital. If the procedure was elective patients were preloaded with 300 mg clopidogrel.

After the procedure, patients were maintained on aspirin 100 mg and clopidogrel 75 mg daily. After BMS implantation clopidogrel was used for 4 weeks, after DES implantation it was used for 6 months, and in case of an acute coronary syndrome it was used for 12 months after the index procedure. Life-long aspirin was prescribed for every patient. Other medications such as ß-Blockers, angiotensin-converting enzyme inhibitors and statins were given as indicated.

Use of glycoprotein IIb/IIIa inihibitors was left to the discretion of the operator. GPIIb/IIIa inhibitors represents a component of standard care at our hospital wherever a large thrombus burden is observed.

During the procedure, intravenous heparin was given to maintain an activated clotting time of > 250 sec. The radial or femoral approach and 6 F guiding catheters were used as a matter of routine.

Our standard procedure (established before the start of this registry) during interventions for bifurcation lesions involved the following: (1) wiring of both branches of the bifurcation lesion, (2) pre-dilatation of the main branch, (3) stenting of the main branch using a DES or a BMS, (4) in case of residual stenosis of the side branch > 50% or a TIMI flow < 2 of the side branch angioplasty or stenting (“provisional stenting”), (5) final kissing wherever a side branch had an angioplasty or stenting. Patients with a diameter of the side branch < 1.5 mm by eye ball were not included.

If a good result was achieved with a TIMI flow III in both branches after stenting of only the main branch, or if there was no visible stenosis of the side branch > 50% and/or the patient had no angina after removal of the balloon from the lesion the procedure was terminated without PCI of the side branch. In this group a really simple concept was scheduled, with no attempted treatment strategy for the side branch. These patients are represented in group A.

A defined treatment strategy for the side branch was prespecified only in patients with the above mentioned criterias (group B, e.g. TIMI flow reduction or a visible stenosis of the side branch > 50%).

A final kiss was attempted for every patient where the side branch was treated, but this was not the case where patients were treated using the simple strategy (group A). The first-line therapy in this case was a simultaneous balloon angioplasty as a “final kissing” PCI. If this strategy could not be accomplished due to failure of balloon placement, the strategy was switched to a sequential balloon angioplasty.

Indications for the placement of a side branch stent in group B included [1] a residual stenosis after balloon angioplasty > 50%, [2] a flow limiting dissection, [3] presence of a thrombus or [4] occlusion of the side branch after balloon angioplasty.

Implantation of additional stents to cover the whole lesion or a dissection were also permitted. However, no combination of drug-eluting stents with bare metal stents was to have been performed.

The balloons were choosen to achieve a balloon-vessel ratio of ~ 1 after measuring the pre-procedure reference vessel diameter. We used semi-compliant balloons with nominal inflation pressures.

Implantation of a drug-eluting stent was preferred in patients with an acute coronary syndrome (NSTEMI and STEMI), in patients with long lesions (> 20 mm) and a small vessel diameter (< 2.5 mm), and in diabetes mellitus patients.

### Angiographic evaluation

Quantitative coronary angiographic (QCA) analysis was performed using Quantcor QCA (version V2.0 by Pie Medical Imaging, Maastricht, The Netherlands). QCA measurements were performed by an independent operator unaware of the details of the therapy using a dye-filled catheter as reference.

The minimal lumen diameter (MLD) and percentage diameter stenosis were measured pre-procedure and post-procedure. The reference vessel diameter of the main branch and the side branch was set up where the diseased segment seems to be unobtrusive.

### Clinical definitions and follow-up

Follow-up was carried out either at an office visit, by looking for data in our local hospital data base or via a telephone call (with the patient or the home physician) where the rate of MACE (major adverse cardiac events) was determined. No scheduled follow-up angiography was indicated unless it was for patients with significant coronary lesions for PCI not related to the target bifurcation lesion, or patients with evidence of disease progression due to a new angina pectoris and/or objective evidence of ischemia.

The follow-up data were defined as either death, myocardial infarction (STEMI and NSTEMI), stent thrombosis, CABG or target lesion revascularization (TLR). All deaths were considered cardiac unless otherwise documented.

The diagnosis of AMI (STEMI or NSTEMI) both peri-procedurally and at follow-up required an elevation of creatine kinase to levels twice those of the upper normal limit together with a rise in the creatine kinase-MB fraction, an elevation of troponin I and/or new ST-segment elevations or new Q-waves (ECG). The threshold used for classifying a positive troponin I test was 0.1 ng/ml. For CK the manufacturer reported a lower threshold of > 2.8 μmol/s/l.

Target lesion revascularization (TLR) was defined as either surgical or percutaneous re-intervention driven by 1) significant (> 50%) luminal diameter narrowing either within the stent or within the 5 mm proximal or distal to the MB or SB stent edge, 2) stent thrombosis or 3) TLR-related CABG. This was undertaken in the presence of either anginal symptoms or objective evidence of an ischemia including stent thrombosis.

Target vessel revascularization (TVR) was defined as revascularization by PCI or surgery within the target vessel encompassing the target lesion including TLR plus PCI target vessel-non target lesion plus CABG including target vessel, not related to the target lesion.

MACE was defined as TVR plus cardiac death.

Classification of a stent thrombosis was based on the definitions of the Academic Research Consortium (ARC) regarding definite, probable or possible stent thrombosis [9]. Stent thromboses were categorized according to the timing of the event into: intraprocedural thrombosis, subacute thrombosis (from the end of the procedure to 30 days), and late stent thrombosis (> 30 days).

### Statistical analysis

95% confidence intervals for the differences between both groups are presented in the tables. For binary variables, odds ratios and their 95% corresponding confidence intervals were calculated using logistic regressions; for ordinate variables, confidence intervals referred to the differences between the means.

For a few variables, as indicated in the tables, log values were taken to accommodate for deviations from normality where the differences in the resulting means were tested using *t*-tests. Statistics from all tests are reported as 2-sided probability values. All calculations were performed using Stata 11.

## Results

During the period between January 2008 and August 2011 we performed 4070 cardiac catheterizations and 1688 percutaneous interventions (41.5%). Of these, 138 patients had a bifurcation lesion (8.2%).

Patients were excluded from analysis who had an in-stent-restenosis (n = 8, 0.5%), who had a drug-coated balloon during PCI (n = 14, 0.8%), and for whom the side branch had not been covered by the stent (n = 18, 1.1%).

**Figure 1:**
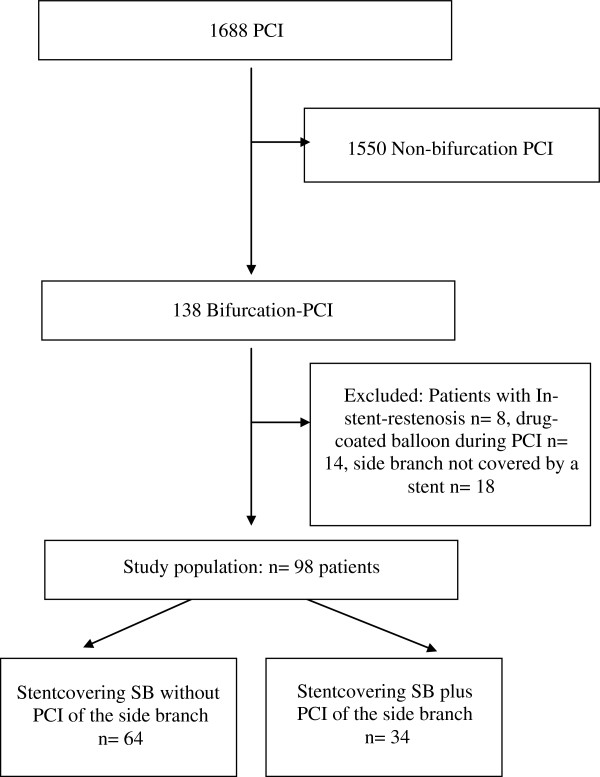
Patient flow.

The rest of the population (n = 98, 5.8%) constituted our study group (see Figure 1).

### Baseline characteristics

Baseline and clinical characteristics (including age, sex, incidence of risk factors) were well matched between the two groups (see Table 1). The mean age was 70.3 years (group A) vs. 67.0 years (group B, p = 0.22) and 65.6% of group A vs. 70.6% of group B (p = 0.62) were male.

**Table 1:** Basic data

	**Covering SB without PCI SB (group A)**	**N**	**Covering SB + PCI SB (group B)**	**N**	**Difference**	**CI; *P***
Age (years: mean, range, standard deviation)	70.3 (40–93; 12.9)	64	67.0 (43–86; 11.8)	34	3.3^b^	(−2.0; 8.6) 0.22
Men (no., percent)	42 (65.6%)	64	24 (70.6%)	34	1.26^a^	(0.51; 3.09) 0.62
Diabetics (no., percent)	14 (21.9%)	64	5 (14.7%)	34	0.62^a^	(0.20; 1.89) 0.40
Diabetics, insulin dependent (no., percent)	5 (7.8%)	64	2 (5.9%)	34	0.74^a^	(0.14; 4.02) 0.73
Hypertension (no., percent)	51 (79.7%)	64	25 (73.5%)	34	0.71^a^	(0.27; 1.88) 0.49
Persistent smoker (no, percent)	11 (17.2%)	64	2 (5.9%)	34	0.30^a^	(0.06; 1.45) 0.13
Hypercholesterolemia (no., percent)	28 (43.8%)	64	14 (41.2%)	34	0.90^a^	(0.39; 2.09) 0.81
Prior PCI (no., percent)	20 (31.3%)	64	11 (32.4%)	34	1.05^a^	(0.43; 2.57) 0.91
Prior CABG (no., percent)	5 (7.8%)	64	1 (2.9%)	34	0.35^a^	(0.04; 3.19) 0.36
NSTEMI (no., percent)	21 (32.8%)	64	9 (26.5%)	34	0.74^a^	(0.29; 1.86) 0.52
STEMI (no., percent)	14 (21.9%)	64	5 (14.7)	34	0.62^a^	(0.20; 1.89) 0.40

^a^ Odds ratio; ^b^ Difference in means.

The cardiac history of the patients regarding prior PCI and CABG was no significantly different between the groups as was the incidence of acute coronary syndrome (NSTEMI and STEMI). An acute coronary syndrome was present in 54.7% (group A) vs 41.2% (group B, see Table 1).

### Lesion characteristics

Details regarding lesion characteristics in the two groups are reported in Tables 2 and 3. Most of the bifurcations were located in the region of the LAD/diagonal branches (59.4% [group A] vs. 55.9% [group B, p = 0.74]), while an angiographically visible thrombus was present in 15.6% (group A) vs. 23.5% (group B, p = 0.36). Both groups did not differ significantly regarding their Medina classifications (Fisher`s exact test: p = 0.09, see Table 3).

**Table 2:** Lesion characteristics

	**Covering SB without PCI SB (n = 64, group A)**	**N**	**Covering SB + PCI SB (n = 34, group B)**	**N**	**Difference**	**CI; *P***
3-vessel disease (no., percent)	19 (29.7%)	64	13 (38.2)	34	1.47^a^	(0.61; 3.52) 0.39
Ejection fraction (%: mean, range, standard deviation)	53.4% (20–70; 11.5)	64	55.7 (40–65; 8.9)	34	−2.4^b^	(−7.4; 2.7) 0.35
Visible thrombus (no, percent)	10 (15.6%)	64	8 ( 23.5%)	34	1.63	(0.58; 4.62) 0.36
Bifurcation localization: LM (no., percent)	4 (6.3%)	64	1 (2.9%)	34	0.62^a^	(0.06; 6.16) 0.68
Bifurcation localization: LAD/SB (no., percent)	38 (59.4%)	64	19 (55.9%)	34	0.87^a^	(0.37; 2.01) 0.74
Bifurcation localization: LCX/SB (no., percent)	15 (23.4%)	64	9 (26.5%)	34	1.18^a^	(0.45; 3.06) 0.74
Bifurcation localization: RCA/SB (no., percent)	7 (10.9%)	64	5 (14.7%)	34	1.40^a^	(0.41; 4.81) 0.59

^a^ Odds ratio; ^b^ Difference in means; ^c^ From a Fisher’s exact test.

**Table 3:** Medina classification

	Covering SB without PCI SB (group A) *	Covering SB + PCI SB (group B) *
Medina classification 1-1-1 (no, percent)	12 (20.0%)	2 (6.5%)
Medina classification 1-1-0 (no, percent)	9 (15.0%)	4 (12.9%)
Medina classification 1-0-1 (no, percent)	5 (8.3%)	8 (25.8%)
Medina classification 0-1-1 (no, percent)	1 (1.7%)	3 (9.7%)
Medina classification 1-0-0 (no, percent)	18 (30.0%)	9 (29.0%)
Medina classification 0-1-0 (no, percent)	14 (23.3%)	5 (16.1%)
Medina classification 0-0-1 (no, percent)	1 (1.7%)	0 (0.0%)
Total	60 (100%)	31 (100%)

* Four patients of group A could not be classified due to complete occlusion of the target vessel in patients with STEMI during non-elective PCI. After restoration of blood flow (thrombectomy or PCI using a undersized balloon) a significant bifurcation lesion was identified.

* Three patients of group B could not be classified due to complete occlusion of the target vessel in patients with STEMI during non-elective PCI. After restoration of blood flow (thrombectomy or PCI using a undersized balloon) a significant bifurcation lesion was identified.

Four patients of group A (6.3%) and three patients (8.8%) of group B could not be classified regarding their Medina classification due to a complete occlusion of the target vessel in patients with STEMI during non-elective PCI. After restoration of blood flow (thrombectomy or PCI using an undersized balloon) a significant bifurcation lesion was identified.

### Procedural details

A thrombectomy was performed before PCI in 9.4% (group A) vs 11.8% (group B, p = 0.71), GP IIb/IIIa antagonists were used in more patients of group B (24.2%) than in group A (14.1%), although this difference was not significant (p = 0.24, see Table 4).

**Table 4:** Procedural details

	**Covering SB without PCI SB (group A)**	**N**	**Covering SB + PCI SB (group B)**	**N**	**Difference**	**CI; *P***
Thrombectomy before PCI (no, percent)	6 (9.4%)	64	4 (11.8%)	34	1.29^a^	(0.34; 4.92) 0.71
Primary stenting (no, percent)	19 (29.7%)	64	8 (23.5%)	34	0.73^a^	(0.28; 1.90) 0.52
Stent diameter (mm, mean, range, SD)	2.7 (2.0 – 3.5, 0.31)	64	2.8 (2.0 – 3.5, 0.38)	34	−0.15^b^	(−0.29; -0.01) 0.04
Stent length (mm, mean, range, SD)	17.6 (8.0 – 58.0, 8.8)	64	20.7 (8.0 – 44.0, 8.8)	34	−0.18^c^	(−0.36; -0.00) 0.05
Drug eluting stent (no., percent)	36 (56.3%)	64	17 (50.0%)	34	0.78^a^	(0.34; 1.79) 0.56
Glycoprotein IIB/IIIA antagonists (no., percent)	9 ( 14.1%)	64	8 (24.2%)	34	1.88^a^	(0.65; 5.43) 0.24
Duration of x-ray, min (mean, range, SD)	18.1 (2.7-220; 28.5 )	64	20.1 (5.8-51.4; 12.2 )	34	−0.36^c^	(−0.67; -0.06) 0.02
Amount of contrast medium, ml (mean, range, SD)	225.8 (70–640; 104.0)	64	307.4 (180–470; 83.9 )	34	−81.5^a^	(−122.8; -40.3) <0.001

^a^ Odds ratio; ^b^ Difference in means; ^c^ Average difference, logged values (for not normally distributed variables).

Sequential or parallel final kissing was performed in n = 14 patients (41.2% in group B). The reasons underlying the PCI of the side branch in group B were the following: stenosis > 50% after covering the side branch (n = 29, 85.3%), closure of the side branch during the procedure (n = 2, 5.9%), and visible thrombus after stent covering (n = 3, 8.8%).

Primary stenting was carried out in 29.7% (group A) vs 23.5% (group B, p = 0.52). The use of drug-eluting stents was homogenously distributed in both groups (56.3% of patients of group A vs. 50.0% of patients of group B, p = 0.56). In group A, we used a paclitaxel eluting stent in n = 17 (26.6%), a zotarolimus eluting stent system in n = 12 (18.8%, Resolute Integrity) and a polymer-free rapamycin eluting stent system (Yukon choice) in n = 7 (10.9%). In group B a paclitaxel eluting stent was used in n = 11 (32.4%), a zotarolimus eluting stent was used in n = 5 (14.7%) and a polymer-free rapamycin eluting stent was used in n = 1 (2.9%).

Interestingly, the stent length was longer in group B (20.7 mm [group B] vs. 17.6 mm [group A]), although this difference just failed to reach significance (p = 0.05). The mean stent diameter was 2.7 mm (group A) vs 2.8 mm (group B), a difference which though small was significant (p = 0.04).

With the simpler strategy, both the amount of contrast medium (ml) and the duration of x-raying (min) were both significantly reduced in group A (group A vs group B, CM usage: 225.8 ml vs. 307.4 ml [p < 0.001] and x ray time : 18.1 min vs 20.1 min [p = 0.02], see Table 4).

### Procedural results

Final TIMI flow III in the main branch was reached in 98.4% vs. 97.1% (group A vs. group B, p = 0.68) while in the side branch it was reached in 84.4% vs. 94.1% (group A vs. group B, p = 0.13, see Table 5).

**Table 5:** Procedural results

	**Covering SB without PCI SB (n = 64, group A)**	**Covering SB + PCI SB (n = 34, group B)**	**Difference**	**CI; *P***
Final TIMI flow III (MB, (no, percent)	63 (98.4%)	33 ( 97.1%)	1.62^a^	(0.16; 16.23) 0.68
Final TIMI flow III (SB, (no, percent)	54 ( 84.4%)	32 (94.1%)	3.32^a^	(0.69; 15.94) 0.13
Final TIMI flow 0 (SB) (no, percent)	5 (7.8%)	0 (0.0%)	0.078^b^	p = 0.16^c^
Final TIMI flow 0 (MB) (no, percent)	0 (0.0%)	0 (0.0%)	0^b^	n.a.
Improvement of TIMI flow ≥ 1 (MB) during PCI (no, percent)	13 (20.3%)	10 (29.4%)	1.63^a^	(0.63; 4.25) 0.31
Improvement of TIMI flow ≥ 1 (SB) during PCI (no, percent)	10 (15.6%)	10 (29.4%)	2.25^a^	(0.83; 6.11) 0.11
Worsening of TIMI flow ≥ 1 (MB) during PCI (no, percent)	0 (0.0%)	0 (0.0%)	0^b^	n.a.
Worsening of TIMI flow ≥ 1 (SB ) during PCI (no, percent)	4 (6.3%)	2 (5.9%)	0.94^a^	(0.16; 5.40) 0.94

^a^ Odds ratio; ^b^ Difference in means.

A final TIMI flow 0 inside the side branch was observed in 7.8% of patients of group A and in no patient of group B (p = 0.16). No patient from either group had a final TIMI flow of 0 inside the main branch.

Interestingly a deterioration of TIMI flow to ≥ 1 in the side branch was present in 6.3% of group A vs 5.9% of group B (p = 0.94), while an improvement of TIMI flow inside the side branch was seen in more patients of group B (29.4%) and in 15.6% of patients of group A, although this difference was not statistically significant (p = 0.11, see Table 5).

### QCA analysis

The QCA analysis of the main branch revealed no significant differences between the groups regarding minimal luminal diameter, the percent diameter stenosis or the lesion length (see Table 6).

**Table 6:** QCA analysis

**Main branch**	**Covering SB without PCI SB (n = 64, group A)**	**Covering SB + PCI SB (n = 34, group B)**	**Difference**	**CI; *P***
Minimal luminal diameter, before/after PCI mm (mean, range, SD)	0.88 (0–1.9; 0.4)	0.82 (0–1.6; 0.4)	0.06^a^	(−0.11; 0.23) 0.46
	2.1 (1.1-3.9; 0.5)	2.0 (1.3-3.4; 0.5)	0.00^a^	(−0.21; 0.21) 0.99
% diameter stenosis before/after PCI (mean, range, SD)	65.4 (48–100; 12.5)	66.3 (49–100; 11.8)	−0.9^a^	(−6.5; 4.7) 0.74
	16.0 (0–44; 10.5)	19.6 (2–44; 11.1)	−3.6^a^	(−8.4; 1.2) 0.14
Reference diameter mm (mean, range, SD)	2.6 (1.3-4.6; 0.6)	2.5 (1.2-4.4; 0.7)	0.07^a^	(−0.23; 0.37) 0.65
Lesion length mm(mean, range, SD)	7.5 (1.5-23.6; 3.5)	9.4 (3.6-31.1; 5.6)	−0.20^b^	(−0.42 ; 0.03) 0.08
Side branch	Covering SB without PCI SB (n = 64, group A)	Covering SB + PCI SB (n = 34, group B)	Difference	CI; *P*
Minimal luminal diameter, before/after PCI mm (mean, range, SD)	1.2 (0–2.5; 0.5)	1.3 (0.3-2.4; 0.6)	−0.08^a^	(−0.33; 0.16) 0.50
	1.1 (0–2,3; 0.6)	1.4 (0.7-2.2; 0.4)	−0.27^a^	(−0.50; -0.03) 0.03
% diameter stenosis before/after PCI (mean, range, SD)	34.2 (0–100; 27.0)	44.0 (12–100; 24.7)	−9.83^a^	(−21.8; 2.2) 0.11
	34.1 (0–100; 25.0)	22.8 (2.9-51.0; 13.5)	11.30^a^	(1.42; 21.17) 0.03
Reference diameter mm (mean, range, SD)	1.8 (0.6-3.3; 0.6)	2.1 (1.3-2.92; 0.5)	−0.28^a^	(−0.53; -0.03) 0.03
Lesion length mm(mean, range, SD)	3.0 (0.0-9.2; 2.1)	3.0 (0.3-9.0; 2.1)	−0.12^b^	(−0.58; 0.34) 0.61

^a^ Difference in means; ^b^ Average difference, logged values (for not normally distributed variables).

The reference diameter of the side branch was significantly larger in group B (2.1 mm vs. 1.8 mm for group B vs group A, p = 0.03), whereas the lesion length was not different.

The minimal luminal diameter and the percent diameter stenosis of the side branch after the intervention was significantly larger in patients of group B (1.1 mm/34.1% [group A] vs. 1.4 mm/22.8% [group b, p = 0.03 for both modalities]) which can be easily explained by the fact that a PCI of the side branch was done in this treatment group (see Table 6).

### Clinical outcome

Target lesion revascularization occurred in 15.9% of patients of group A and in 29.4% of patients in group B (p = 0.12), whereas the incidence of target vessel revascularization was 15.9% (group A) vs. 32.4% (group B, p = 0.07, see Table 7). No myocardial infarction (except stent thrombosis) was observed in either group.

**Table 7:** Follow-up data (multiple statements were possible)

	**Covering SB without PCI SB (group A)**	**N**	**Covering SB + PCI SB (group B)**	**N**	**Difference**	**CI; *P***
Re-PCI MB, In-segment (no., percent)	5 (7.9%)	63	4 (11.8%)	34	1.55^a^	(0.39; 6.19) 0.54
Re-PCI SB, In-segment (no., percent)	3 (4.8%)	63	3 (8.8%)	34	1.94^a^	(0.37; 10.16) 0.44
Definite stent thrombosis ( no., percent)	0 (0%)	63	3 (8.8%)	34	−0.088^b^	0.041^c^
CABG related to the target lesion (no., percent)	2 (3.2%)	63	0 (0%)	34	0.032^a^	0.54
TLR (no., percent)	10 (15.9%)	63	10 (29.4%)	34	2.21^a^	(0.81; 6.01) 0.12
PCI target vessel, non-target lesion or CABG not related to target lesion (no., percent)	0 (0%)	63	1 (2.9%)	34	−0.029^b^	0.35^c^
TVR (no., percent)	10 (15.9%)	63	11 (32.4%)	34	2.53^a^	(0. 95; 6.80) 0.07
Cardiac death (no., percent)	5 (7.9%)	63	5 (14.7%)	34	2.00^a^	(0.54; 7.47) 0.30
All MACE (no., percent)	15 (23.8%)	63	16 (47.1%)	34	2.84^a^	(1.17; 6.92) 0.02
PCI non-target vessel (no., percent)	6 (9.5%)	63	5 (14.7%)	34	1.64^a^	(0.46; 5.82) 0.45
Diagnostic cardiac catheterization without PCI (no., percent)	3 (4.8%)	63	2 (5.9%)	34	1.25^a^	(0.20; 7.87) 0.81
Non-cardiac death (no., percent)	2 (3.2%)	63	0 (0%)	34	0.032^b^	0.54^c^
Lost of follow-up (no., percent)	1 (1.6%)	64	0 (0%)	34	−0.016^b^	1.00^c^
Follow-up duration, months (mean, range, SD)	14.1 (0–41; 13.1)	63	12.3 (1–46; 12.8)	34	1.79^b^	(−3.70; 7.27) 0.52

^a^ Odds ratio; ^b^ Difference in means; ^c^ From a Fisher’s exact test.

The occurrence of a definite stent thrombosis was 0% in group A and 8.8% (n = 3) in group B (p = 0.04). Two of these patients experienced this event, one on day 3 and the other on day 8 after the index procedure under aspirin plus clopidorel, with these events being suffered as a subacute thrombosis according to the definition of the Academic Research Consortium (ARC). One patient was treated with aspirin monotherapy and suffered an incident on day 472 after the PCI. As such he was defined as having suffered a late stent thrombosis.

A (Re)-PCI of the side branch during follow up was seen in 4.8% (group A) vs 8.8% (group B, p = 0.44, see Table 7) of the patients.

One very important issue was the analysis of the nature of cardiac death. Cardiac death was observed in 5 patients from each group (group A = 7.9%, group B = 14.7%, p = 0.30).

Sudden cardiac death was observed in n = 4 patients from group A (6.4%) with these events occurring away from the hospital on day 3, 7, 30 and 61 after the index procedure: all these patients were under combined therapy with aspirin plus clopidogrel. These events had to be classified as a possible stent thrombosis according to the ARC definitions [9]. One patient (1.5%) of group A died in our hospital on day 8 due to progressive heart failure.

All patients from group A who suffered a cardiac death during follow-up were usually admitted to our clinic before the index PCI with an acute coronary syndrome (NSTEMI or STEMI).

The analysis of the patients from group B revealed the following: sudden cardiac death occurred in one patient on day 229 (2.9%) under aspirin plus clopidogrel (this had to be defined as a possible stent thrombosis, see ARC definitions [9]); one patient (2.9%) had a definite stent thrombosis (verified during cardiac catheterization) on day 7 under combined anticoagulation with aspirin and clopidogrel; one patient (2.9%) died due to progressive heart failure 4 months after PCI; one patient (2.9%) was admitted to our hospital with a STEMI and resuscitation before arrival in our clinic and died after development of cardiogenic shock on day 3; one patient (2.9%) died of unknown cause, the cause could have not been classified (this was regarded as cardiac death).

Four of these patients were primarily (before the index PCI) were admitted to the hospital due to an acute coronary syndrome (NSTEMI and STEMI).

An investigation of all MACE revealed a significant difference in favour of group A (23.8% [group A] vs. 47.1% [group B, p = 0.02].

## Discussion

The major findings of the present study were that: 1) the MACE rate in patients with de novo bifurcation lesions, stent covering of the side branch and PCI of the side branch is significantly higher than in patients without PCI of the side branch, 2) cardiac death tended to show a higher incidence in patients where the side branch was treated, especially in patients who were initially admitted with an acute coronary syndrome (NSTEMI or STEMI), 3) the functional results looking at TIMI flow inside the main branch and the side branch showed no significant differences between the two therapeutic modalities 4) the duration of x-raying and the amount of contrast medium was significantly lower in patients with only PCI of the main branch.

How can these results be interpreted? The basic data from the two groups revealed no significant differences, and there were also no significant differences regarding lesion characteristics and the procedural details. Thrombectomy, drug-eluting stents and GPIIb/IIIA antagonists were all used to an almost identical extent in the two groups.

Interestingly, the stent length in the patients from group B was slightly longer (20.7 mm vs. 17.6 mm, p = 0.05) which may have contributed to a higher incidence of consecutive stent thrombosis and sudden cardiac death. Consistently with this we found a higher incidence of definite stent thrombosis amongst group B patients.

In patients with an acute coronary syndrome, a significantly higher amount of contrast medium for complex and long-lasting interventions might trigger pump failure and cardiogenic shock, a scenario which might explain the different MACE rates that were observed.

Other procedural artifacts (stent deformation, minimal disruption with subclinical dissections) for patients having undergone the complex procedure might also have contributed to these results since manipulations with the guide wire had to be carried out for longer.

In patients treated with a complex strategy for coronary artery bifurcation lesions other observers [6] have noted a higher incidence of cardiac biomarkers release and a higher level of contrast medium usage. This may have contributed to a higher incidence of MACE, in analogy with our own results.

The observed MACE rate in our study group was consistent with other studies including patients with an acute coronary syndrome [1]. In comparison to Adriaenssens et al. [1] we observed a higher incidence of patients with an acute coronary syndrome.

Our own group [10] were able to show that a flow-guided treatment concept for the side branch is not inferior compared to a more complex strategy where the standard routine was to treat both the main branch and the side branch. Our actual results showed that this seems to be the concept of choice for patients undergoing an elective procedure.

In a group of patients with a high incidence of acute coronary syndromes such as that seen in our study population, a strictly conservative procedure would appear to be superior where the primary goal of therapy is treatment of the main branch. A side branch PCI should only be indicated in patients where a large side branch becomes occluded, and only if the amount of contrast medium used is strictly controlled. Anatomic details of a residual stenosis > 50% in the side branch like as was our initial strategy for the therapyof de-novo bifurcation lesions seems to be less relevant.

### Clinical implications

We conclude that a simple strategy for patients with de-novo coronary bifurcation lesions where the side branch is covered with a stent shows better long-term results when the side branch is not treated (under the assumption that the treatment strategy was a result of the angiographic view). This may be an important issue especially for patients presenting with acute coronary syndromes.

To the best of our knowledge this study is the first where harm has been documented as a result of using complex bifurcation treatment strategies.

### Study limitations

In our study we a systematic approach for all patients presenting with a coronary bifurcation lesion. As well as all of these patients we also included those presenting with acute coronary syndromes.

Nevertheless, our database represents a single-center retrospective analysis that was not randomized, a fact which also has to be kept in mind. The patients and operators were not blinded to the different treatment modalities.

## Conclusions

In patients with coronary bifurcation lesions, a simpler strategy involving no SB-PCI results in a significantly lower MACE rate compared to a strategy involving treatment of the SB.

## Competing interests

The authors declare that they have no competing interests.

## Authors’ contributions

The authorship included the following: HVK, VS, KC, BS, JH: conception and design of data, analysis and interpretation of the results. RVE: statistical analysis, data analysis and interpretation of the results. UH, MO: drafting of the manuscript, critically revising. BL, TM: final approval of the manuscript. All authors read and approved the final manuscript.

## Pre-publication history

The pre-publication history for this paper can be accessed here:

http://www.biomedcentral.com/1471-2261/13/27/prepub
